# Revisiting minimally important changes for the Oxford Hip and Knee scores

**DOI:** 10.1186/s41687-026-01024-1

**Published:** 2026-02-20

**Authors:** Adam B. Smith, Damian Lewis, Stuart Mealing, Andria Joseph

**Affiliations:** York Health Economics Consortium, University of York, York, YO10 5NQ UK

## Abstract

**Purpose:**

A number of measures have been proposed to evaluate meaningful within-person change in the Oxford Hip and Oxford Knee scores (OHS and OKS), however there is evidence of lower baseline scores being associated with higher change scores, that is, these instruments potentially demonstrate baseline dependency. The study aim was to identify and quantify the impact of baseline dependency for the OHS and OKS.

**Patients & methods:**

The data were collated from the National Health Service in England including the OHS, OKS, EQ-5D-3L and a global transition item (GTI). Change scores, including the minimally important change (MIC) were derived and categorised by the GTI and baseline scores for the OHS and OKS. Baseline dependency was evaluated using different baseline categories (OHS/OKS, EQ-5D quartiles and split-item method).

**Results:**

A total of 387,524 records were extracted. Although the overall MIC were in-line with previous research, the results showed these measures varied by pre-operative scores. Baseline dependency was present irrespective of the method employed to categorise change scores.

**Conclusions:**

The MICs for both the OHS and OKS show distinct baseline dependencies. The use of a single MIC for either instrument is unlikely to capture the full range of meaningful change experienced by individual patients and therefore has implications for the interpretation of interventional outcomes with these instruments. A multifaceted approach involving multiple sources of patient-relevant measures is recommended to provide more robust measures for the evaluation of patient outcomes and healthcare services.

**Supplementary Information:**

The online version contains supplementary material available at 10.1186/s41687-026-01024-1.

## Introduction

Patient-reported outcome (PRO) measures directly capture aspects of individuals’ health-related quality of life (HRQoL) as reported by patients themselves [1] and play an important role in determining what matters most to patients in the management of their health. The Oxford Hip (OHS) and Oxford Knee Scores (OKS) [2, 3] were developed to measure function and pain following hip and knee replacement. These measures have been used extensively in clinical research, have demonstrated robust psychometric properties, including internal consistency, construct and content validity [4] and are routinely employed in the UK National Health Service (NHS) to evaluate patient outcomes and quality of care following surgery [5].

An important aspect of this evaluation involves interpreting the meaningfulness of change in PRO scores. The minimally important change (MIC) is a measure that may assist in this process. Although there are different definitions [6], central to the MIC concept is that it reflects the smallest beneficial change perceived by the individual [7]. Critically, the MIC refers to within-person change over time perceived to be important by the individual patient [8], and is a key psychometric property related to an instrument’s ability to detect change [8].

Several studies have derived MIC estimates for the OKS and OHS [9, 10]. These range from 9 to 12 for the OHS for evaluating, respectively, either primary hip replacement or revision surgery, and for the OKS between 9 and 11 [9, 10]. More recent research has developed categories for interpreting clinically meaningful change in the OKS [11] with change scores *≥* 16 indicating significant improvement (“much better”) in patients’ subjective rating of their health and scores between 7 and 15 a moderate improvement (“little better”). The same study also determined higher boundary scores dependent on baseline scores with the thresholds between categories increasing to *≥* 18 and 9–17 (thresholds between, respectively, “a little” / “much better”, and “about the same” / “a little better”) for those patients with baseline scores less than the mean OKS (< 19). The latter study indicates that baseline scores may impact on the MIC.

In addition to these measures of MIC for the OHS and OKS, post-operative success has also been defined either in terms of score categories, e.g., “excellent” or “good” outcome [12–14] or score thresholds beyond which surgical outcomes could be deemed to be successful [15–17]. Composite score thresholds including multiple patient-reported evaluations of surgical success have also been shown to vary with baseline scores [16].

Several studies have therefore suggested a potential baseline dependency for the OHS and OKS. Baseline dependency, where the minimally important change is dependent on the baseline PRO score, has been observed across other medical conditions and other PROs [18–20]. These findings suggest that those patients with poorer baseline health states require greater levels of improvement in health to report important change [21]. Given the use of MICs in general, and specifically the use of the OHS and OKS in evaluating meaningful change in clinical trials, research, and clinical practice it is important to determine the relationship between baseline and change scores.

The purpose of this study was therefore to further clarify meaningful change associated with the OHS and OKS specifically to determine whether these PROs exhibit baseline dependency and if so, to assess how this dependency impacts on change scores, particularly the MIC.

## Methods

### Data and instruments

The data were collated from publicly available data published between 2015 and 2022 [5]. These data were originally collected as part of the UK’s National Health Service Patient Reported Outcome Measures Programme (NHS PROMS) [5]. Patients undergoing elective hip or knee replacement procedures (either total hip or total knee arthroplasty) are invited to complete both a condition-specific (Oxford Hip or Oxford Knee Score) and a generic PRO (the EQ-5D-3 L ) [22]. The PROs are completed pre-surgery as well as up to 6 months post-surgery. Although patient completion of the PROS is voluntary, all healthcare organisations providing NHS-funded hip and knee replacement surgery are mandated to collect these data [5]. These data are used, amongst others, by NHS providers to benchmark performance against national averages with the aim of improving care quality.

The Oxford Hip and the Oxford Knee Scores (OHS; OKS) [2, 3] are two 12-item condition-specific PROMS designed to provide patient self-report of their health status and benefits of treatment. The items in each PRO describe the level of pain, limitations on daily activities and self-care, mobility resulting from the patient’s hip or knee condition.

The 5 response categories for the individual items for these PROs are reverse scored from 4 (“None” or “No”) to 0 (“Severe”, “Impossible”, “Unbearable”). A higher score reflects better function (maximum score: 48).

The EQ-5D-3L [22] is a generic PRO with 5 single-item domains: mobility, self-care, usual activities, pain/discomfort, anxiety/depression. The 3-point response categories (no problems, moderate problems, severe problems) for each item are converted into health utilities, a measure of health-related quality of life (HRQoL), using a country-specific algorithm. Health utilities range from 0 “dead”) to 1 (“perfect health”). Health states worse than dead (< 0) are also possible. The UK algorithm [22] is used by the NHS PROMS Programme for conversion of the patient responses into health utilities.

In addition to the PROs, patients also rate post-surgery improvement in their hip/knee on a 5-point global transition item (GTI: “much better”; “a little better”; “about the same”; “a little worse”; and “much worse”).

### Statistical analysis

A basic summary of patient baseline characteristics was derived (count and percentages). Summary statistics were created for the OHS and OKS scores (mean and standard deviation). Additionally, an overall mean change score (difference between post- and pre-operative PRO scores) was derived as well as the standard deviation of the change. These were also calculated for each category on the global transition item (GTI) for each PRO. Following previous research [9] the minimally important change (MIC) was designated as the mean change from baseline for those patients reporting “a little better” on the GTI. The change scores for both PROs were categorised by baseline scores as follows using previously published categories: baseline scores *≤* 19; *≥*20 and *≤* 29; *≥* 30 and *≤* 39; and > 40 [12, 15, 16].

The degree of measurement error of a PRO may be estimated using the standard error of measurement (SEM). Meaningful change needs to be evaluated against the SEM, to separate out true change from measurement error. The minimal detectable change (MDC) represents the smallest change that can be considered to be distinct from measurement error. In line with previous research on the OHS and OKS [8], the MDC was derived from the SEM as follows:

$$\begin{aligned}&\rm SEM = {SD_{change}}^* \sqrt (1-internal \ reliability), and\cr&\:\mathrm{M}\mathrm{D}\mathrm{C}\:=\:\surd\:2\:\mathrm{*}\:1.65\:\mathrm{*}\:\mathrm{S}\mathrm{E}\mathrm{M}\end{aligned}$$

Given the overall patient scores on the OHS and OKS were expected to change between pre- and post-surgery, and furthermore allowing for the length of time between assessments (6 months), the internal reliability (Cronbach’s alpha) was employed to calculate the SEM. The overall standard deviation of change scores (SD_change_) was also applied for each PRO. The 1.65 multiple reflects the z-value for the 90% confidence level [9].

Previous literature has indicated that the MIC should be greater than 4 times the SEM in order to distinguish the MIC metric from measurement error [23]. The 4SEM criterion was calculated using data from those patients recording no change in their health (“about the same”) [23]. The 4SEM criterion and MDC were used to evaluate meaningful improvement.

Previous research [11] has demonstrated an association between baseline scores on the OKS and the level of minimally important change, i.e., patients with lower baseline OKS required greater levels of change to record an MIC. Therefore, patients in the current study were categorised into two groups based on the mean baseline OKS. Evidence suggests that stratifying on baseline scores may introduce bias and consequently result in spurious baseline-dependency [24]. Three alternative methods have been proposed as alternatives to determine baseline-dependency [24]. These include using the PRO in question for a second, independent baseline assessment (“Method 1”). Applying a different PRO (highly correlated with the original PRO) to provide baseline stratification (“Method 2”) or splitting the original PRO into two parallel item sets and estimating the MIC by stratifying on one set of baseline scores (and vice versa) (“Method 3”) [24]. Given no additional PRO data were available only Methods 2 and 3 were applied.

For Method 2, the level of association between EQ-5D scores and the OHS and OKS were calculated using Pearson Product-Moment correlation. The OHS and OKS change scores (and MIC) were stratified using the baseline EQ-5D-3L scores categorised into quartiles. For Method 3 (split-item method), a random set of 6 items were selected for each PRO to generate one set (“Form A”) with the remaining set of 6 items used for the second set (“Form B”). Total scores were calculated for each form for both pre- and post-surgery OHS and OKS scores. The baseline scores for each form were then stratified into quartiles. Subsequently, change scores for each PRO were derived and categorised using Form B baseline categories for the Form A items, and a second set were derived using Form A baseline categories for the Form B items.

Finally, to further investigate any potential baseline dependencies the mean change score was derived for each baseline score for both the OHS and OKS in order. This was achieved by treating each baseline score (0, 1, 2…48) as an individual factor and deriving the corresponding change score to provide a mean change score for each baseline score. The results of this analysis were summarised graphically.

The analysis was undertaken using R version 4.4.2.

## Results

### Participants

A total of 490,574 patients underwent hip replacement surgery, and 552,959 patients underwent knee replacement surgery during the period 2015 to 2022. Of these patients, 407,564 (83%, hip) and 476,211(86%, knee) completed the PROs prior to surgery; 271,785 (67% of those completing the OHS pre-surgery) and 309,137 (65% of those completing the OKS pre-surgery) also completed the PROs at follow-up post-surgery. The overall participation rate for this period was therefore 55% and 56% respectively for hip and knee surgery. The extracted data for this study comprised a total of 184,509 patients who had completed the OHS and 203,015 who had completed the OKS both pre- and post-surgery. This represents 68% (hip) and 66% (knee) of those patients with complete data for both assessments (and respectively 38% and 37% of all patients undergoing either hip or knee replacement surgery in this time period).

An overview of the patients’ sociodemographic details is provided in Table 1. Females represented 56% (hip) and 52% (knee) of the sample. Two-thirds of the hip replacement patients were aged between 60 and 79 years; this figure was 75% for patients undergoing knee surgery. Pre-operative physical disability was reported by half the sample. In terms of pre-existing medical conditions, the majority of patients reported arthritis (73% and 78% for hip and knee). Other comorbidities included high blood pressure (38% and 44%), diabetes (9% and 12%), pulmonary disease (8% and 9%), and depression (8% and 9%).

**Table 1:** Patient sociodemographic and basic clinical details

	Hip surgery patients	Knee surgery patients
**Total**	*N* = 184,509	*N* = 203,015
**Age band (years)(** ***N*** **,%)**		
*	11,955 (6%)	11,396 (6%)
30 to 39	48 (0%)	-
40 to 49	2,436 (1%)	257 (0%)
50 to 59	22,747 (12%)	20,894 (10%)
60 to 69	57,219 (31%)	68,840 (34%)
70 to 79	67,558 (37%)	79,974 (39%)
80 to 89	22,464 (12%)	21,654 (11%)
90 to 120	82 (0%)	-
**Gender (N**,**%)**		
*	11,955 (6%)	11,396 (6%)
Male	69,817 (38%)	85,179 (42%)
Female	102,737 (56%)	106,440 (52%)
**Previous surgery (N**,**%)**		
Yes	14,849 (8%)	15,002 (7%)
No	168,608 (91%)	186,863 (92%)
Not recorded	1,052 (1%)	1,150 (1%)
**Pre-operative co-morbidities**		
**Physical Disability (N**,**%)**		
Yes	90,642 (49%)	95,838 (47%)
No	84,935 (46%)	100,374 (49%)
Not recorded	8,932 (5%)	6,803 (3%)
**Arthritis (N**,**%)**		
Yes	134,045 (73%)	157,972 (78%)
No	50,464 (27%)	45,043 (22%)
**High Blood Pressure (N**,**%)**		
Yes	69,362 (38%)	89,919 (44%)
No	115,147 (62%)	113,096 (56%)
**Diabetes (N**,**%)**		
Yes	16,767 (9%)	24,868 (12%)
No	167,742 (91%)	178,147 (88%)
**Pulmonary disease (N**,**%)**		
Yes	15,065 (8%)	18,282 (9%)
No	169,444 (92%)	184,733 (91%)
**Depression (N**,**%)**		
Yes	15,216 (8%)	18,582 (9%)
No	169,293 (92%)	184,433 (91%)
**Heart Disease (N**,**%)**		
Yes	15,433 (8%)	18,523 (9%)
No	169,076 (92%)	184,492 (91%)
**Cancer (N**,**%)**		
Yes	10,248 (6%)	10,990 (5%)
No	174,261 (94%)	192,025 (95%)
**Circulation problems (N**,**%)**		
Yes	7,801 (4%)	10,247 (5%)
No	176,708 (96%)	192,768 (95%)
**Nervous system (N**,**%)**		
Yes	1,505 (1%)	2,027 (1%)
No	183,004 (99%)	200,988 (99%)
**Liver disease (N**,**%)**		
Yes	1,185 (1%)	1,178 (1%)
No	183,324 (99%)	201,837 (99%)
**Stroke (N**,**%)**		
Yes	2,349 (1%)	3,150 (2%)
No	182,160 (99%)	199,865 (98%)

*Numbers not provided for small hospitals in order to preserve patient anonymity

### The Oxford Hip Score (OHS)

The mean baseline score for OHS was 18 (Standard deviation, SD: 8.32) and 40 (SD: 8.57) at follow-up. Cronbach’s alpha (internal reliability) for the OHS was 0.89. The SEM was 3.35, MDC 7.81; and 4*SEM for the OHS was 13.4.

### Minimally important change: Oxford Hip Score

The mean overall change score (Table 2) for the OHS was 22.01 (SD: 10.13). The OHS change scores increased in line with the GTI categories from 0.60 (SD: 11.34) for “much worse” to 23.81 (SD: 8.88) for “much better.” The overall MIC for the OHS was 12.56 (rounded-up to 13).

**Table 2:** Mean change from baseline in Oxford Hip & Knee Scores by baseline categories and global transition item

Oxford Hip Score Baseline / GTI	*N*	Overall	Much worse	Little worse	About the same	MIC / Little better	Much better
≤ 19	109,817	26.09 (9.36)	3.2 (10.5)	6.5 (7.7)	10.5 (8.6)	15.3 (7.6)	28.1 (7.6)
≥ 20 & ≤29	57,033	18.02 (7.25)	-1.8 (10.8)	0.9 (7.3)	5.6 (7.9)	9.5 (6.7)	19.5 (5.7)
≥ 30 & ≤39	16,040	10.34 (6.28)	-7.6 (10.5)	-3.8 (7.1)	1.0 (6.8)	4.0 (6.2)	11.7 (4.7)
≥ 40	1,619	1.22 (6.49)	-20.2 (9.0)	-7.8 (7.3)	-3.1 (5.7)	-2.0 (6.9)	2.9 (4.6)
Total	184,509	22.01 (10.13)	0.60 (11.34)	3.13 (8.53)	7.68 (8.93)	12.56 (8.22)	23.81 (8.88)

Oxford Knee Score Baseline / GTI	N	Overall	Much worse	Little worse	About the same	MIC/ Little better	Much better
≤ 19	106,656	20.42 (9.86)	0.7 (7.4)	5.0 (6.4)	8.0 (6.9)	13.4 (6.9)	24.3 (7.3)
≥ 20 & ≤29	75,501	14.60 (7.78)	-4.0 (8.7)	0.6 (6.4)	3.8 (6.5)	8.4 (6.0)	17.2 (5.7)
≥ 30 & ≤39	19,723	8.12 (6.70)	-9.2 (8.3)	-3.5 (6.6)	-0.6 (6.2)	3.1 (5.8)	10.2 (4.8)
≥ 40	1,135	0.99 (6.44)	-16.9 (10.5)	-8.5 (7.0)	-6.8 (6.4)	-3.1 (5.8)	2.9 (4.3)
Total	203,015	16.95 (9.78)	-1.49 (8.55)	2.59 (7.05)	5.85 (7.30)	10.84 (7.35)	20.00 (8.14)

The baseline category analysis showed that the overall level of mean change, 26.09 (SD: 9.36) was greatest for those patients with baseline / pre-surgery OHS *≤* 19 and smallest for those patients with baseline OHS scores *≥* 40 (mean OHS: 1.22 (SD:6.49)). This was also observed for each GTI category with mean change score decreasing as baseline OHS scores increased and was also notable for the MIC which ranged from 15.3 (SD: 7.6) to -2.0 (SD: 6.9) for those baseline categories suggesting baseline-dependency.

### Evaluating OHS baseline-dependency: method 2

The correlation (rho) between the OHS and EQ-5D pre-operative scores was 0.73 (95% Confidence Interval, CI: 0.729; 0.734, *p* < 0.001). The correlation coefficient indicated good association between the measures underlining the suitability of the EQ-5D for the baseline stratification. The pre-operative EQ-5D scores quartiles for the OHS were: <0.055; *≥* 0.055 & *≤* 0.52; >0.52 & *≤*0.69; and > 0.69.

The overall mean OHS change scores (Supplementary Table 1a) decreased from 26.5 (SD: 10.4) for patients with the lowest EQ-5D Index score category (0.055) to 16.9 (SD: 8.7) for the highest baseline score category (EQ-5D Index > 0.69). The MIC, similarly, also decreased from 15.5 (7.9) to 8.6 (7.7) across the baseline EQ-5D Index categories. Although the pattern of scores was similar to those observed in the stratification by baseline OHS scores, the mean MIC values tended to be higher across the EQ-5D baseline categories (particularly for EQ-5D scores *≥* 0.055).

### Evaluating OHS baseline-dependency: method 3

The results of the Method 3 analysis (Supplementary Tables 2a and 2b) showed that both the overall change scores for the OHS (on both forms) decreased in line with increasing baseline. These results were also observed for the MIC which decreased from 8 to 3.

### Summary for the Oxford Hip Score MIC

All three methods applied demonstrated a baseline dependency between the MIC and baseline scores, irrespective of the method applied to stratify the pre-operative OHS scores.

### Range of MIC for the Oxford Hip Score

**Fig. 1:**
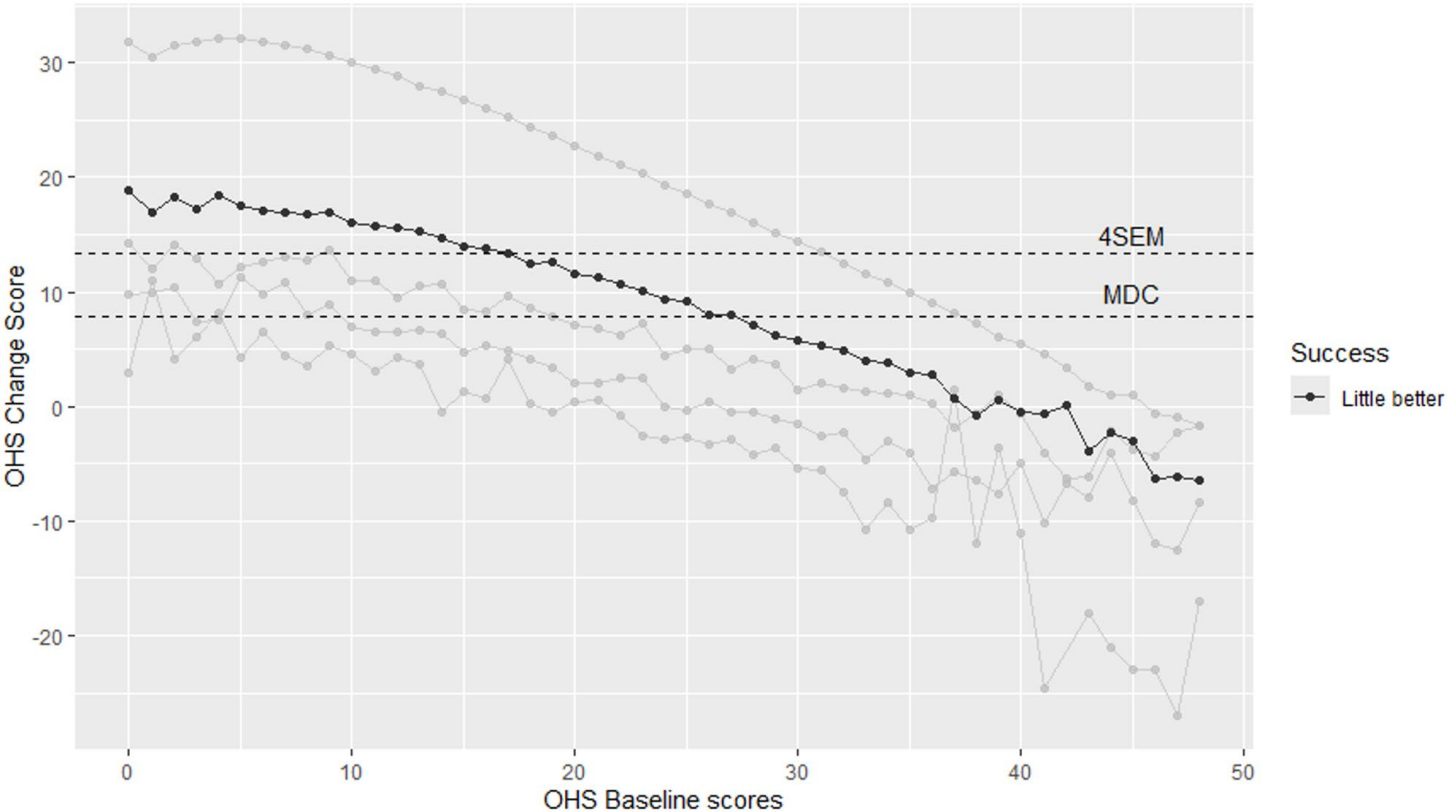
Oxford hip score by baseline scores

The OHS baseline analysis is summarised in Fig. 1, which shows the mean OHS change score plotted against the OHS baseline scores. The MIC has been highlighted (“A little better”). This shows an approximately (negative) linear relationship between the MIC and baseline OHS scores with MIC values decreasing as baseline OHS scores increase. This adds further evidence underlining the baseline-dependency of the MIC for the OHS. Lines have been included in the figure highlighting the MDC and 4SEM.

### The Oxford Knee Score (OKS)

#### Minimally important change: Oxford Knee Score

The mean baseline score for OKS was 18 (Standard deviation, SD: 8.32) and 40 (SD: 8.57) at follow-up. The test-retest reliability (rho) of the OKS for those patients reporting no change in their health (“About the same”) was 0.57. Cronbach’s alpha (internal reliability) for the OKS was 0.88. The SEM was 3.43, MDC 7.99; and 4*SEM for the OKS was 13.7.

#### Minimally important change: Oxford Knee Score

The mean overall change score for the OKS was 16.95 (SD: 9.78) (Table 2). As seen with the OHS, the mean OKS change scores increased in line with the GTI categories from − 1.49 (SD: 8.55) for “much worse” to 20.00 (SD: 8.14) for “much better”. The overall MIC for the OKS was 10.84 (rounded up to 11).

The overall level of mean change for the OKS when stratified by baseline OKS scores was highest at 20.42 (SD: 9.86) for those patients with baseline / pre-surgery OKS *≤* 19. The smallest overall OKS change score, 0.99 (SD: 6.44) was observed for those patients with baseline OKS scores *≥* 40. In line with the results from the OHS, the mean change decreased for each baseline category consistently with increasing baseline OKS scores. This was also shown for the MIC which ranged from − 3.1 (SD: 5.8) for baseline OKS scores *≥* 40 to 13.4 (SD: 6.9) for baseline scores *≤* 19. Again, these results indicate baseline-dependency.

### Evaluating OKS baseline-dependency: method 2

The correlation coefficient (rho) for the OKS and EQ-5D pre-operative scores was 0.70 (95%CI: 0.696; 0.701, *p* < 0.001) suggesting the EQ-5D was appropriate for the baseline stratification. The pre-operative EQ-5D scores quartiles for the OKS were: <0.10; *≥* 0.10 & *≤* 0.59; >0.59 & *≤*0.69; and > 0.69.

The results are shown in Supplementary Table 1b. The overall OKS mean change score decreased from 20.0 (SD: 10.6) to 11.4 (SD: 8.3) as the baseline EQ-5D score categories increased. Furthermore, the MIC also demonstrated baseline-dependency, decreasing from 13.5 (SD: 7.1) to 6.0 (SD: 7.0) in line with increases in baseline EQ-5D scores., similarly, also decreased from 15.5 (7.9) to 8.6 (7.7) across the baseline EQ-5D Index categories. As with the OHS change scores, the OKS changes seen with the EQ-5D baseline categories were higher than those observed for the baseline OKS categories but nevertheless indicate baseline-dependency.

### Evaluating OKS baseline-dependency: method 3

The results of the Method 3 analysis (Supplementary Tables 3a and 3b) showed that both the overall change scores for the OKS (on both forms) decreased in line with increasing baseline, although the changes Form B (Supplementary Table 3a) were slightly higher than those observed on Form A (Supplementary Table 3).

### Summary for the Oxford Knee Score MIC

The results for the OKS across the three methods showed baseline-dependency between the MIC and baseline scores.

### Range of MIC for the Oxford Knee Score

**Fig. 2:**
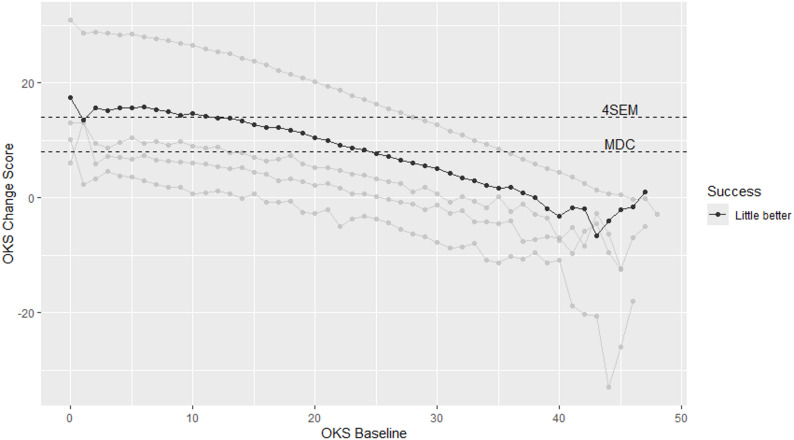
Oxford Knee Score by baseline scores

The mean OKS change score plotted against the OKS baseline scores is shown in Fig. 2 with the MIC highlighted (“A little better”). The results demonstrate a (negative) linear relationship between the MIC and baseline OKS scores, in other words, the MIC values decrease as baseline OKS scores increase, indicating baseline-dependency for the OKS.

## Discussion

The aim of the study was to determine whether there were baseline dependencies for the OHS and OKS in terms of the change scores, and in particular, the MIC. A secondary aim to this was to evaluate how change scores might vary by baseline score.

Firstly, the minimal detectable change (MDC), the threshold beyond which “real” change may be said to have occurred rather than measurement error, was around 8 for both the OHS and OKS. Although the MDC does not reflect meaningful change it may be used as a benchmark to evaluate meaningful change scores [8]. The results for the overall MIC for both instruments, respectively 13 and 11 (OHS and OKS) were in-line with previous research [9, 10] and exceeded the MDC, but only the MIC for the OHS equalled the associated 4*SEM criterion (13). The OKS MIC fell slightly short of the 4*SEM criterion (14).

In terms of the relationship between baseline scores and change scores: The results showed a clear baseline dependency for both instruments. Both the overall mean change scores and the MICs decreased as baseline category scores increased. These results were maintained irrespective of the methodology applied [24]. It should be noted that change scores were higher for both the OHS and OKS when stratified using the baseline EQ-5D scores, especially for EQ-5D categories beyond the worst baseline scores (EQ-5D scores < 0.055 and < 0.10, respectively). This may reflect a less than perfect correspondence between the EQ-5D and OHS/OKS. Nevertheless, a clear baseline dependency was observed for the EQ-5D stratification, as well as for the split-item stratification (Method 3). Furthermore, given the results held across the different methods applied it may be concluded that the baseline dependencies are not an artefact of the stratification process and reflect a real correspondence between baseline and (meaningful) change scores.

Previous research on the OKS had shown that pre-operative scores may affect the level of MIC when applying a mean-split [11]. The current study extends these results both for the OKS as well as the OHS. In addition, as well establishing baseline dependencies for both PROs, the results also demonstrated a negative linear relationship between baseline scores and the mean change score (for each baseline score). This also showed, for instance, that only those patients with baseline scores *≤* 27 for the OHS or *≤* 20 for the OKS reported change scores greater or equal to the MDC. It should also be noted that the MICs when stratified by baseline categories (for the OHS/OKS as well as EQ-5D scores) only exceeded the respective 4*SEM criteria when the OHS/OKS baseline scores were *≤* 19 (and lowest EQ-5D score category).

These results underline research that has previously highlighted the correspondence between baseline scores and the magnitude of change scores [18–20]. This literature has suggested that baseline dependency arises due to the fact that those patients in poorer health states require a greater degree of change (in the health) [21, 24] in terms of rating an improvement of their health, relative to those in better health states. This has the concomitant effect of larger MICs for lower baseline scores as evidenced in this study.

In terms of the implications of the results, the MIC, that is minimally important within-person change, may be used for different purposes. One of which may be as a measure in evaluating the number of patients achieving the MIC or “responders” in a given trial (and thereby determining the success or otherwise of a trial) or for instance in clinical practice following the introduction of certain treatments [8, 25, 26]. The results of this study indicate a range of change scores or MICs depending on the individual patient’s baseline score. In the context of clinical practice, where baseline scores and change scores will be available this may aid clinician-patient interactions. However, in the case of clinical trials, the range of MICs may present issues in determining the number of responders. In instances such as these, it may be more fruitful for researchers to determine the number of patients recording at least some or a little improvement in their health state (i.e., a category analysis), rather than focusing on the magnitude of change.

The study results chime with recent calls to move away from single scale-specific MICs [27] in the use of trial design and evaluating outcomes in clinical research, as well as advocating a more holistic approach based on a triangulation of multiple methods [28].

The main study limitation is the fact that no direct interpretation of patient-reported change in health was available with the analysis solely reliant on a single item response. Nevertheless, this should be balanced in mitigation against the large sample sizes.

## Conclusion

The MICs for both the OHS and OKS show distinct baseline dependencies. The use of a single MIC for either instrument is unlikely to capture the full range of meaningful change experienced by individual patients and therefore has implications for the interpretation of interventional outcomes or clinical research with these PROs. A multifaceted approach involving multiple sources of patient-relevant measures is recommended to provide more robust measures for the evaluation of patient outcomes and healthcare services.

## Supplementary Information

Below is the link to the electronic supplementary material.

Supplementary Material 1

## Data Availability

The datasets analysed during the current study are available in the NHS Digital repository, https://digital.nhs.uk/data-and-information/data-tools-and-services/data-services/patient-reported-outcome-measures-proms.
