# Supplementary material for: Revisiting minimally important changes for the Oxford Hip and Knee scores
Source: J Patient Rep Outcomes. 2026 Feb 20;10:45. doi: 10.1186/s41687-026-01024-1 (PMC13031445; doi:10.1186/s41687-026-01024-1)
Supplement: Supplementary file 1 — Supplementary Material 1 [file 41687_2026_1024_MOESM1_ESM.docx]

**Supplementary Table 1a. Oxford Hip Scores – Change from baseline categorised by EQ-5D (Method 2)**

| **OHS change scores / EQ-5D baseline categories** | **Overall** | **Much worse** | **Little worse** | **About the same** | **MIC / Little better** | **Much better** |
| --- | --- | --- | --- | --- | --- | --- |
| <0.055 | 26.5 (10.4) | 3.0 (9.3) | 6.5 (7.6) | 10.3 (8.6) | 15.5 (7.9) | 29.0 (8.5) |
| >0.055 & <0.516 | 23.9 (9.6) | 1.6 (11.3) | 4.6 (8.5) | 8.9 (8.7) | 13.4 (7.9) | 25.8 (8.0) |
| >0.52 & <0.69 | 20.1 (8.9) | -1.5 (11.5) | 1.8 (7.6) | 6.6 (8.5) | 10.9 (7.7) | 21.7 (7.5) |
| >0.69 | 16.8 (8.7) | -3.1 (12.8) | -0.7 (8.4) | 4.2 (8.5) | 8.6 (7.7) | 18.2 (7.5) |

**Supplementary Table 1b. Oxford Knee Scores – Change from baseline categorised by EQ-5D (Method 2)**

| **OKS change scores / EQ-5D baseline categories** | **Overall** | **Much worse** | **Little worse** | **About the same** | **MIC / Little better** | **Much better** |
| --- | --- | --- | --- | --- | --- | --- |
| <0.10 | 20.0 (10.5) | 0.6 (6.8) | 5.0 (6.3) | 7.8 (6.8) | 13.5 (7.1) | 24.5 (8.0) |
| >0.10 & <0.59 | 18.6 (9.8) | -0.7 (8.9) | 3.2 (7.0) | 6.4 (7.4) | 11.4 (7.1) | 22.0 (7.6) |
| >0.59 & <0.69 | 15.4 (8.6) | -3.8 (8.9) | 1.2 (6.8) | 4.7 (6.9) | 9.3 (6.8) | 17.9 (6.9) |
| >0.69 | 11.4 (8.3) | -5.7 (9.5) | -1.5 (7.3) | 1.8 (7.3) | 6.0 (7.0) | 13.5 (6.9) |

**Supplementary Table 2a. Oxford Hip Scores – Baseline subset categories (Method 3) – Form A baseline and Form B change scores**

| **OHS change scores / Subset Baseline** | **N** | **Overall** | **Much worse** | **Little worse** | **About the same** | **MIC / Little better** | **Much better** |
| --- | --- | --- | --- | --- | --- | --- | --- |
| <6 | 46,128 | 14.1 (5.4) | 2.3 (5.1) | 4.0 (4.4) | 5.8 (4.7) | 8.2 (4.3) | 15.2 (4.5) |
| >6 & <9 | 46,127 | 12.8 (4.9) | 1.7 (5.9) | 2.6 (4.2) | 5.1 (4.7) | 7.4 (4.2) | 13.7 (4.0) |
| >9 & <12 | 46,127 | 11.0 (4.5) | -0.3 (5.4) | 1.7 (4.3) | 3.9 (4.4) | 6.3 (4.1) | 11.8 (3.8) |
| >12 | 46,127 | 7.7 (4.5) | -2.8 (6.1) | -1.0 (4.4) | 1.5 (4.4) | 3.7 (4.2) | 8.5 (3.9) |
| **Total** | 184,509 |  |  |  |  |  |  |

**Supplementary Table 2b. Oxford Hip Scores – Baseline subset categories (Method 3) – Form B baseline and Form A change scores**

| **OHS change scores / Subset Baseline** | **N** | **Overall** | **Much worse** | **Little worse** | **About the same** | **MIC/**  **Little better** | **Much better** |
| --- | --- | --- | --- | --- | --- | --- | --- |
| <5 | 46,128 | 13.5 (5.2) | 2.0 (5.5) | 3.9 (4.3) | 5.7 (4.6) | 8.0 (4.2) | 14.6 (4.3) |
| >5 & <8 | 46,127 | 11.9 (5.2) | 0.5 (5.4) | 2.4 (4.1) | 4.4 (4.4) | 6.8 (4.0) | 12.8 (3.8) |
| >8 & <12 | 46,127 | 10.1 (4.3) | 0.1 (5.6) | 1.2 (3.9) | 3.4 (4.4) | 5.5 (3.9) | 10.9 (3.6) |
| >12 | 46,127 | 7.0 (4.2) | -2.4 (5.7) | -0.6 (4.2) | 1.5 (4.0) | 3.4 (3.7) | 7.8 (3.6) |
| **Total** | 184,509 |  |  |  |  |  |  |

**OHS items in Form A: Pain, Washing, Dressing, Walking, Standing, Work;**

**OHS items in Form B: Sudden Pain, Night Pain, Transport, Shopping, Limping, Stairs.**

**Supplementary Table 3a. Oxford Knee Scores – Baseline subset categories (Method 3) – Form A baseline and Form B change scores**

| **OKS change scores / Subset Baseline** | **N** | **Overall** | **Much worse** | **Little worse** | **About the same** | **MIC / Little better** | **Much better** |
| --- | --- | --- | --- | --- | --- | --- | --- |
| <6 | 50,754 | 11.4 (5.6) | 0.9 (4.3) | 3.2 (4.2) | 5.0 (4.2) | 8.1 (4.3) | 13.7 (4.3) |
| >6 & <9 | 50,754 | 10.1 (5.0) | -0.3 (4.7) | 2.4 (4.1) | 4.0 (4.1) | 6.9 (4.0) | 11.8 (3.8) |
| >9 & <12 | 50,754 | 8.7 (4.6) | -1.1 (5.2) | 1.5 (4.1) | 3.3 (4.2) | 5.8 (3.9) | 10.0 (3.6) |
| >12 | 50,753 | 6.3 (4.3) | -2.7 (5.3) | 0.1 (4.1) | 1.7 (4.1) | 3.8 (3.8) | 7.4 (3.6) |
| **Total** | 203,015 |  |  |  |  |  |  |

**Supplementary Table 3b. Oxford Knee Scores – Baseline subset categories (Method 3) – Form B baseline and Form A change scores**

| **OKS change scores / Subset Baseline** | **N** | **Overall** | **Much worse** | **Little worse** | **About the same** | **MIC/**  **Little better** | **Much better** |
| --- | --- | --- | --- | --- | --- | --- | --- |
| <7 | 50,754 | 9.3 (5.4) | 0.3 (3.2) | 2.1 (2.9) | 3.3 (3.3) | 5.7 (3.5) | 11.5 (4.4) |
| >7 & <10 | 50,754 | 8.7 (5.0) | -0.5 (3.9) | 1.2 (3.2) | 2.6 (3.4) | 5.1 (3.5) | 10.4 (4.0) |
| >10 & <13 | 50,754 | 7.6 (4.6) | -1.8 (4.2) | 0.4 (3.3) | 1.8 (3.5) | 4.1 (3.5) | 9.0 (3.8) |
| >13 | 50,753 | 5.6 (4.5) | -3.4 (4.6) | -1.0 (3.6) | 0.4 (3.6) | 2.6 (3.5) | 6.8 (3.7) |
| **Total** | 203,015 |  |  |  |  |  |  |

**OKS items in Form A: Pain, Transport, Standing, Kneeling, Shopping, Stairs.**

**OKS items in Form B: Night Pain, Washing, Walking, Limping, Work, Confidence.**
